# Effectiveness of Gutting Blue Whiting (*Micromesistius poutassou*, Risso, 1827), in Spanish Supermarkets as an Anisakidosis Safety Measure

**DOI:** 10.3390/foods10040862

**Published:** 2021-04-15

**Authors:** Ana Elena Ahuir-Baraja, Lola Llobat, Maria Magdalena Garijo

**Affiliations:** 1 Parasitology and Parasitic Disease Research Group (PARAVET), Department of Animal Production and Health, Public Veterinary Health and Food Science and Technology, Faculty of Veterinary Medicine, Universidad Cardenal Herrera-CEU, CEU Universities, Calle Tirant lo Blanc, 7, 46115 Alfara del Patriarca, Spain; ana.ahuir@uchceu.es; 2 Microbiological Agents Associated with Animal Reproduction Research Group (PROVAGINBIO), Department of Animal Production and Health, Public Veterinary Health and Food Science and Technology, Faculty of Veterinary Medicine, Universidad Cardenal Herrera-CEU, CEU Universities, Calle Tirant lo Blanc, 7, 46115 Alfara del Patriarca, Spain

**Keywords:** *Anisakis*, blue whiting, muscle, gutting, *Hysterothylacium*, safety

## Abstract

Anisakidosis is a parasitic zoonotic disease which can cause gastroallergic reactions in humans. In 2010, the European Food Safety Agency estimated that approximately 20,000 cases of anisakiasis had been reported across the world, with Spain having the highest number of infections in Europe. The blue whiting (*Micromesistius poutassou*, Risso, 1827) is one of the most widely fished species worldwide and represents around 25% of the white fish eaten in Spain. The Spanish Food Safety Authority requires obligatory evisceration of certain fish species before commercialization, but not for blue whiting. Nevertheless, some supermarkets carry this out themselves to prevent human infections and negative customer reactions deriving from the presence of ascaridoid larvae. To assess the effectiveness of eviscerations at supermarkets, a total of 320 blue whiting specimens were examined. The risk of larval migration from the visceral cavity to the musculature in gutted and ungutted fish was also assessed. Our results showed a total prevalence (25%) of ascaridoids in fish gutted at the supermarket, and a direct relationship was found between the presence of larvae in the muscle and time until evisceration. In ungutted fish, the standard length and weight were higher for infected than for non-infected fish. Also, massive infections had a higher prevalence in these larger specimens, in which the viability of larvae was also high. Larval viability was not found to be affected by a 24-h refrigeration period. *Anisakis* was the most prevalent genus identified in the fish examined. The results indicate that gutting at the supermarket is not an effective method for the total removal of ascaridoid larvae and that additional safety measures are advisable.

## 1. Introduction

Anisakidosis is a fish-borne zoonosis caused by ascaridoid nematodes belonging mainly to the Anisakidae family Railliet and Henry, 1912. It is a major emerging human disease worldwide, with humans acting as accidental hosts [1,2,3,4]. The genera causing human infection are *Anisakis*, *Contracaecum*, *Pseudoterranova* and, less usually, *Hysterothylacium*, this last belonging to the family Raphidascarididae [4,5,6,7,8,9,10]. The genus *Anisakis* is the most frequently reported, and the infection with this genus being known as anisakiasis [8]. Human infection occurs when viable third stage larvae are accidentally ingested in raw or undercooked fish and cephalopods, in forms such as sashimi, sushi, ceviche, cold smoked fish, or marinated fish [11]. Gastroallergic anisakiasis has been described as the most frequent type of reaction [5,12,13,14]. It is caused by hypersensitivity to larval proteins and symptoms include hives and anaphylactic shock [15,16]. Allergic reactions have even been described after the consumption of previously frozen or cooked fish, since sensitized patients can suffer responses to dead larvae [12]. Commercial fish-derived products may also provoke allergic anisakiasis [17,18,19], and a recent systematic review has emphasized the need to consider it a public health problem [20].

Anisakidosis cases have increased in recent years all over the world as a result, mainly of improvements in diagnostic methods and the traditional preparations, and to a lesser extent, of changes in gastronomic culture and the increase in world trade [21]. In 2010, the European Food Safety Authority (EFSA, 2010) estimated that approximately 20,000 anisakiasis cases had been reported across the world, with more than 90% of these from Japan. In Europe, Spain has reported the highest number of cases of anisakiasis in humans, and these continue to increase [2,22]. Anisakiasis is now considered to be an occupational disease in Spain, with the presentation of type I hypersensitivity -mainly to *Anisakis* spp.—in individuals who handle fish, such as fishmongers, supermarket employees or chefs [2,23,24]. The higher incidence in Spain can be chiefly attributed to the country’s culinary traditions, with most infections deriving from the popular consumption of European anchovies (*Engraulis encrasicolus*) and European pilchard (*Sardina pilchardus*), marinated in vinegar [2,22]. Other fish which are widely consumed in Spain include blue whiting (*Micromesistius poutassou*), horse mackerel (*Trachurus trachurus*), European hake (*Merluccius merluccius*), and Atlantic bonito (*Sarda sarda*), and these present high infection rates for ascaridoid larvae [25,26,27,28,29,30,31].

The blue whiting, *Micromesistius poutassou* (Risso, 1927) (Gadiformes: Gadidae), is one of the most widely caught fish species worldwide and represents around 25% of the white fish consumed in Spain [31] due to its low cost and its wide availability. In this pelagic fish, the genera *Anisakis*, *Contracaecum*, *Hysterothylacium*, and *Pseudoterranova* have been reported [5,32]. For the genus *Anisakis*, prevalence oscillates from 65 to 99.5 % for the area north of Spain [25,29,33].

A large number of commercial fish species are highly infected. Consequently, prevention measures have been developed to avoid human infection [32,33]. However, consumer confidence is damaged when larvae in fish are found, affecting sales and potentially leading to important economic losses for the fishing sector [34]. On the one hand, the presence of parasites may depress demand for fish but, on the other, parasite elimination procedures also increase the final price [35].

The Spanish Agency for Food Safety and Nutrition (AESAN) includes the gutting of certain fish species as an obligatory procedure before commercialization (AESAN, 2005), although blue whiting is not included in this group. Thus, in some wholesale fish markets *M. poutassou* is gutted by fishmongers before sale as a particular measure.

This study assesses the effectiveness of the gutting procedures used in local supermarkets of Spain for blue whiting in removing ascaridoid larvae. In addition, the effect of a 24-h refrigeration period after purchase on larval viability and migration to muscle is evaluated. The possible presence of visceral remains after gutting and massive larval infection are also noted and commented on. A correlation between the standard length and weight of the analyzed fish, and the presence of larvae in the visceral cavity and muscle is also considered. A morphological identification, at the genus level, of the collected ascaridoid larvae is included.

## 2. Materials and Methods

### 2.1. Sample and Data Collection

A total 344 blue whiting from the North-East Atlantic (the Bay of Biscay off the Spanish coast, fishing area 27.8 according to the FAO, Food and Agriculture Organization of the United Nations), were randomly bought from two local supermarkets in Valencia, Spain. Following the labels, fish for this study had reached the coast the day before the purchase. At the laboratory, freshness was evaluated using parameters such as the turgidity of the eyeball, changes in skin coloration, scale adhesion, color of the gills, odor and, for ungutted fish, the texture and appearance of the visceral organs were checked [36]. After excluding fish with non-integral structure from the study (*n* = 24), a total of 320 blue whiting was examined between February and April of 2019. Two general categories were established, comprising 120 gutted fish (gutted at the supermarket) and 200 ungutted fish (which were examined for parasitological survey before being gutted in the laboratory). Two subgroups were established for each category: fish examined within 24 h of purchase (103 from the gutted category and 119 from the ungutted category), and fish examined 24 h after purchase (17 from the gutted category and 81 from the ungutted category). These latter subgroups were kept refrigerated at 6ºC until the parasitological examination.

The fish were examined and the presence of larvae in the visceral cavity assessed according to routine visual examination for infection with zoonotic nematode larvae with the aid of a stereoscopic microscope (M 205 C, Leica, CA, USA) [37]. The abdomen was cut open using scissors from the anus to the area between the pelvic fins and the outer layer of all internal organs were thoroughly visually examined for the presence of parasites. The digestively tract and swim bladder were cut open and observed with the naked eye ad under a stereoscope. Afterwards, the muscle of each fish was examined down to the final millimeter (including the belly flaps) under a stereoscopic microscope using a scalpel and tweezers. The larvae recovered were placed in a warm saline solution (0.9% NaCl) on Petri dishes and viability was recorded following Codex Stan 244 (2004). Those that moved spontaneously or in response to stimulation with dissecting needles were considered alive. The presence or absence of visceral remains was recorded in the gutted fish group and the standard length and weight of the fish were recorded only for the ungutted category.

For both subgroups in the ungutted fish category, the larvae present in the viscera were counted. When the number of larvae was higher than 30, this was recorded as a massive infection. All larvae collected were fixed and preserved in 70% ethanol for their further identification [38].

### 2.2. Larvae Quantification and Identification

The prevalence and mean abundance (± standard deviation) and 95% confidence interval of the ascaridoid larvae found were calculated [32]. One hundred larvae were randomly selected from the four subgroups, isolated in Petri dishes with warm saline solution (0.9%NaCl), cleared with lactophenol for 24 h and observed under a light microscope (Leica DM 750) for morphological identification to genus level [39,40,41,42]. *Anisakis* spp. Larvae were identified as Type I or II (*sensu* [39]).

### 2.3. Statistical Analysis

The statistical analysis was performed using R statistical software (version 3.6.1) and the Rcmdr package, freely available on CRAN. Categorical factors were analyzed using Pearson’s χ^2^ test and Fisher’exact tests. The confidence intervals for prevalence estimates were calculated using the Wilson score interval method. Quantitative factors were analysed with a non-parametric test (Wilcoxon-Mann Whitney test) to determine differences between categories. Correlations between quantitative factors were analysed using Spearman’s correlation. The Shapiro-Wilk test for normality and Levene’s test for homoscedasticity were used to detect significant difference among group variances. Results were expressed as mean and the confidence interval at 95% for levels of infection and weight and length as mean standard deviation has been showed. The statistical significance was set at *p*-value < 0.05.

## 3. Results

**Figure 1:**
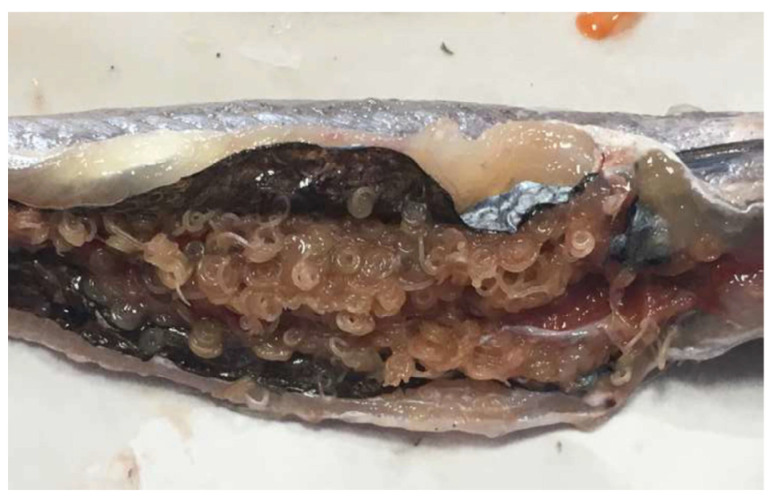
Massive infection of ascaridoid larvae in a blue whiting (*Micromesistius poutassou*).

The overall prevalence of ascaridoid larvae in the 320 blue whiting specimens examined was 45.6% massive infection (i.e., more than 30 larvae being present) was found in 59 of these infected fish (Figure 1). The overall prevalence of infected fish with larvae in the muscle was 25.9%.

In the ungutted category, the prevalence of parasitization was 58%. Of these infected fish, massive infection was present in 47.4% (*n* = 55), with the mean abundance in the other 61 being 2.9 ± 0.4. In the under-24-h subgroup (i.e., those examined within 24 h of purchase), 52.9% were infected. Of these, 39.7% (*n* = 25) presented larvae in the muscle, mainly in the belly flaps. In the post-24-h subgroup, parasites were found in 65.4%, with 33 of these having parasites in the muscle.

**Figure 2:**
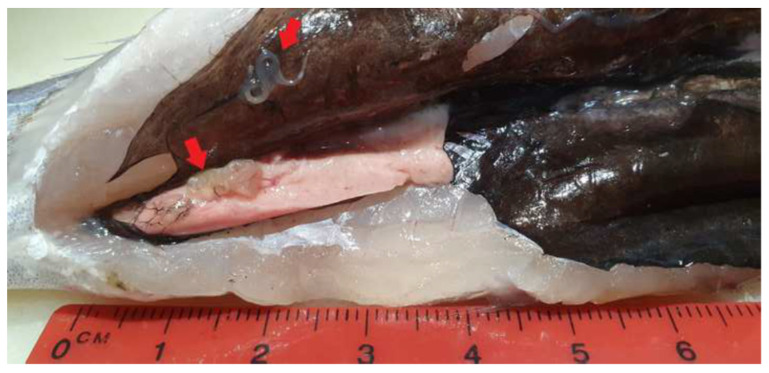
Visceral remains with ascaridoid larvae in a blue whiting gutted at the supermarket.

Of the 120 blue whiting gutted at the supermarket, 10 showed remains of viscera, in most of cases the peritoneum and liver, with the macroscopically visible presence, in all cases, of mobile nematodes and adhered coiled and encapsulated larvae (Figure 2).

In this category, the prevalence of ascaridoid larvae was 25% (*n* = 30). Four of the specimens presented massive infection, with mean abundance in the muscle for the other 26 infected being 5.0 ± 1.1. In the under-24-h subgroup, prevalence was 23.3%, with 79.2% of the infected fish presenting larvae in the muscle. Of the over-24-h subgroup, 35.2% of the fish were infected, with 100% of them showing larvae in the muscle (*n* = 6). In both gutted and ungutted fish, prevalence in the muscle for the under-24-h subgroup was lower, but these differences were only statistically significant for the ungutted category (Table 1). Both in the under-24-h and in the over-24-h subgroups, infection in muscle was similar in ungutted and gutted groups (21 vs. 18.4, and 40.7 vs. 35.3, respectively). The total prevalence of ascaridoid larvae in ungutted fish was higher than in gutted, both in the over-24-h and in the under-24-h (52.9 vs. 23.3, and 65.4 vs. 35.3, respectively) (Table 1) (*p* < 0.05).

In the ungutted fish, relationship between standard length and weight, and infection is shown in Table 2.

**Figure 3:**
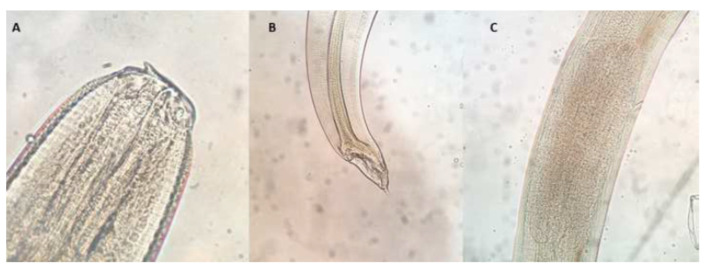
Anterior (**A**), posterior (**B**) and ventricular (**C**) region of an *Anisakis* spp. L3.

The total number of larvae found in 69 fish form the under-24-h subgroups without massive infection was 83.8%. In the over-24-h subgroups, 82 or the 103 larvae recovered from 18 fish remained viable. Differences in viability related to time were not statistically significant. Ninety-seven of the one hundred larvae selected were morphologically identified as *Anisakis* Type I and three as third-stage larvae of *Hysterothylacium* spp. (Figure 3).

## 4. Discussion

This study provides data about the effectiveness of the gutting procedures used by local supermarkets in Valencia, to prevent damage to consumer confidence and infection by ascaridoid larvae in blue whiting, a fish species with a high prevalence of parasitization worldwide. We expected our results to be similar to those reported from other regions in the Iberian Peninsula, since gutting procedures and the origin of the fish are usually the same.

The prevalence of ascaridoid larvae infection found in ungutted blue whiting was higher than those reported in Mediterranean regions of Spain [33,43,44,45,46], and yet lower than those reported in previous studies focusing on the country’s Atlantic coast [25,43,45,47]. Nevertheless, our results confirm the higher prevalence of ascaridoid larvae in fish caught around the Atlantic coasts compared to those from the Mediterranean. This is consistent with the findings of Roca-Geronès et al., who demonstrated that geographical origin is a factor in the abundance of *Anisakis* spp. in blue whiting [29]. This high prevalence is also consistent with data found in the same area for other fish species such as cod (*Gadus morhua*) in recent years [48,49].

The detection procedure is another factor that may influence the prevalence reported. Some authors recommend methods such as artificial digestion or incubation [37,50,51]. These techniques can be useful for the examination of larger species such as cod or hake, which often present high larval infection in the muscle, or for inspecting a large number of specimens in an industrial setting. In our study, given that blue whiting is a small fish species, we decided to make a destructive direct visual inspection and the muscle of each fish was examined down to the final millimeter under a stereomicroscope with a scalpel and tweezers, in order to detect as many larvae as possible. For Llanera-Reino et al. in the absence of a gold standard, visual inspection is the current recommended procedure for anisakid detection and counting in certain fish species [52,53].

Regarding the parasitic burden, our results show a high proportion of massive infections in ungutted fish, which is consistent with previously reported results [54]. This finding is of great importance, because in these case parasites can be macroscopically viewed on the external surface of the fish even through the skin and muscle, leading to negative consumer reactions. Furthermore, the risk of zoonosis when consuming such specimens is higher. Nevertheless, it seems that the presence of visible larvae in the gut does not necessarily imply that they will also be present in the musculature or the edible parts of the fish. No significant relationship was found between the number of parasites in the gut and of those in the muscle of blue whiting in the survey carried out by Llanera-Reino et al. in 2012 [52].

A significant relationship was found between the length and weight of blue whiting and the number of larvae found, with higher burdens observed in larger and heavier fish. These results are consistent with previous studies [25,43,46]. It is known that anisakis larvae are long-lived parasites that tend to accumulate in adult fish, which feed more frequently and usually on larger prey, making them more vulnerable to infection [55,56]. As remarked above, the season of capture seems to be a factor for the number of infections. In 2018, Molina-Fernández et al. [33] fund that blue whiting captured in spring had a higher prevalence for anisakid infection than in autumn, probably due to the larger size of fish caught in this season.

Regarding the morphological identification of the larval ascaridoid found, only two genera were identified in the blue whiting studied here, *Anisakis* and *Hysterothylacium*. The former was found much more frequently, as was the case in previous studies carried out in Spain [25,46]. The zoonotic potential of ascaridoid larvae is widely acknowledged, but controversy exists concerning that of *Hysterothylacium* spp., although this may derive from differences between species [51]. Some studies have observed a lack of resistance of *Hysterothylacium* spp. larvae to digestion [45], although allergic reactions [57] and even human clinical cases related to this nematode have been reported [10]. Interestingly, our findings contrast with the trend observed in other parts of the world. For example, in Australian waters, the population of *Anisakis* is declining and *Hysterothylacium* is increasing [51]. Mattiucci et al. reported that the *Anisakis* population in Norway are shifting towards the Arctic and stated that the infection levels of ascaridoid species in a particular geographic area may be strongly affected by the population size of the host involved in the life cycle [58].

Our findings suggested that gutting procedures were generally carried out correctly at the supermarket, since a low number of fish presented remaining viscera. However, it is important to highlight the risk of anisakidosis and potential damage to consumer confidence that these specimens present, since all of theme possessed macroscopically visible mobile nematodes and coiled and encapsulated larvae in the peritoneum and the remains after evisceration, and even massive infection (more than 30 larvae) in some specimens, as previous studies have found [45,54]. Llanera-Reino et al. found larvae in the liver and gonads from gutted European hake, even after washing with tap water [52].

The time elapsed since the arrival of the fish at the point of distribution until gutting takes place is also an important factor, since in the over-24-h subgroups the total prevalence and the prevalence of fish with larvae in the muscle was higher than in the under-24-h subgroups, indicating the movement of parasites from the viscera to the muscle, using up their energy reserves until death. Post-mortem larval migrations to the muscle were also noted in blue whiting by some authors [29,52,54]. This occurs due to changes in temperature during and after the capture and handling of the fish, causing the activation and movement of the larvae from the visceral cavity to the muscle [59,60]. However, as we have seen, larvae were also found in the muscle even in fresh blue whiting, as has also been reported previously, suggesting *Anisakis* spp. larvae probably also migrate to the musculature in *M. poutassou* while the host is alive [45,52,61]. This may be due to differences in the expression of genes involved in the parasites’ migration and survival through the host tissue, facilitating escape from host reactions [62].

As expected, larval viability in our study was not affected by refrigeration, and consequently it can be said that the risk of human infection remains after one day in a domestic refrigerator (at 6 °C). Despite this, a high percentage of larvae can resist temperature stress and lack of food, due to their ability to synthesize glycogen and trehalose in addition to other molecular mechanisms [63,64]. General recommendations for the prevention of anisakidosis usually insist on the need for an appropriate heat treatment (freezing or cooking) before consumption [65]. However, Podolska et al. demonstrated that freezing temperatures only kill *Anisakis* spp. larvae in certain species of fish [34], and to the authors’ knowledge, freeze resistance studies have not yet been carried out on blue whiting. Control measures should also include training for fishermen to avoid discarding the viscera at sea, in order to prevent further spreading of the parasites in the environment, and a system for the on-board processing of the viscera of fish infected by anisakids has been proposed [66].

## 5. Conclusions

The results of this study show that the gutting carried out at supermarkets in Spain is not an effective method for the total removal of ascaridoid larvae in blue whiting, even if it does considerably reduce the larval burden and the possibility of negative consumer reactions, as well as human gastric and allergic disease. Moreover, the time until gutting procedures are carried out is directly proportional to the probability of finding live larvae in the fish muscle. Larger specimens are more likely to be infected and show a higher larval burden, both in the visceral cavity and in the muscle. Information should be made available to workers and consumers to ensure more exhaustive gutting is carried out and that the gutted fish are then washed with tap water. Additional safety measures, such as placement of labels warning consumers of the possible risks associated with highly-infected species, such as European hake or blue whiting, should be considered in supermarkets. Furthermore, a warning that the size and the origin of the fish is important should be made and, finally, it may even be advisable for allergic consumers to avoid consuming those fish species with a high risk of ascaridoid infection.

## Figures and Tables

**Table 1:** Total prevalence (number of infected blue whiting, *Micromesistius poutassou* compared to the total number of fish examined for each subgroup) and mean abundance of ascaridoid larvae (number of larvae found in fish without massive infection divided by the number of fish examined in each subgroup) in all fish analyzed and in muscle (mean abundance was calculated only in fishes without massive infection). The percentages represent the proportion of infected fish to the total number of examined fish. * *p*-value < 0.05 was considered significant. “ns” = not statistically significant.

					Prevalence of Ascaridoid Larvae in Muscle			Total Prevalence of Ascaridoid Larvae		
Categories and Subgroups		Number of Fish Examined	Mean Length of Fish Examined ±SD (cm)	Weigh of fish Examined ±SD (g)	Mean Abundance in Fishes without Massive Infection (IC 95%)	Prevalence (%)	χ2 (*p*-Value)	Mean Abundance in Fishes without Massive Infection (IC 95%)	Prevalence (%)	χ2 (*p*-Value)
Ungutted fish	<24 h since purchase	119	20.9 ± 2.1	54.3 ± 13.4	0.1 (0.0, 0.2)	21	5.9 (<0.05) *	1 (0.7, 1.3)	52.9	3.1 (ns)
	24 h since purchase	81	22.3 ± 2.6	64.9 ± 21.6	0.2 (0.0, 0.4)	40.7		1.7 (0.6, 2.8)	65.4	
Gutted fish	<24 h since purchase	103	21.6 ± 2.3	46.3 ± 12.8	0.9 (0.3, 1.4)	18.4	1.5 (ns)	1 (0.4, 1.6)	23.3	1.1 (ns)
	24 h since purchase	17	23.2 ± 2.5	56.7 ± 24.0	1.5 (−1.3, 4.4)	35.3		2.2 (−1.3, 5.8)	35.3	
Overall		320	21.5 ± 2.1	48.9 ± 17.3	0.1 (0.1, 0.2)	25.9		0.3 (0.2, 0.4)	45.6	

**Table 2:** Standard length (cm) and weight (g) of infected and non-infected fish (A and B, respectively), and fish with massive infection and without massive infection (C and D, respectively). The data are presented as mean ± SD. * *p*-value < 0.05 was considered significant.

	Standard Length (cm) (Mean ± SD)	*p*- Value	Weight (g) (Mean ± SD)	*p*-Value
Infected fish	22.5 ± 2.7	<0.05 *	65.2 ± 20.3	<0.05 *
Non-infected fish	20.3 ± 1.0		49.5 ± 7.4	
Massive infection	24.7 ± 1.8	<0.05 *	80.7 ± 16.0	<0.05 *
No massive infection	20.5 ± 1.7		51.2 ± 11.9	

## Data Availability

Not applicable.
